# A new classification for septal perforation and effects of treatment methods on quality of life^☆^

**DOI:** 10.1016/j.bjorl.2018.06.003

**Published:** 2018-07-17

**Authors:** Emrah Sapmaz, Yuksel Toplu, Battal Tahsin Somuk

**Affiliations:** a Gaziosmanpaşa University Medical Faculty, Department of Otorhinolaryngology, Tokat, Turkey; b Inonu University Medical Faculty, Department of Otorhinolaryngology, Malatya, Turkey

**Keywords:** Septal perforation Quality of life, Septal button, Perfuração septal, Qualidade de vida, Botão septal

## Abstract

**Introduction:**

Septal perforation is a condition characterized by loss of cartilage and/or bony structures along with the mucoperichondrium and mucoperiosteum lining them. The etiology includes a history of nasal surgery or trauma, nose picking, bilateral septal cauterization, overuse of nasal sprays, cocaine abuse, vasculitis, and malignancies.

**Objective:**

Comparison of quality of life in patients with septal perforation after conservative or surgical treatment, and a new approach for the determination of the diameter of the perforation from a different point of view.

**Methods:**

The diameter of septal perforation, total vertical diameter of septum, and horizontal diameter of the perforation were measured in a total of 34 patients. Nineteen of the patients underwent surgical septal perforation repair, and 15 of them received septal button application. The patients were asked to complete the Glasgow Benefit Inventory quality of life questionnaire.

**Results:**

The septal perforation successfully healed in 18 of 19 patients who underwent surgical treatment. The quality of life scores were statistically significantly higher in the surgical treatment group when compared to the button group (*p* < 0.05).

**Conclusion:**

The septal perforation classification we propose would be beneficial for providing realistic dimensions, treatment methods, and surgical techniques.

## Resumo

**Introdução:**

A perfuração septal é uma condição caracterizada pela perda de estruturas cartilaginosas e/ou ósseas, juntamente com o mucopericôndrio e o mucoperiósteo que as revestem. A etiologia inclui um histórico de cirurgia nasal ou trauma, cutucar o nariz, cauterização septal bilateral, uso excessivo de sprays nasais, abuso de cocaína, vasculite e neoplasias malignas.

**Objetivo:**

Comparar a qualidade de vida em pacientes com perfuração septal após tratamento conservador ou cirúrgico e uma nova abordagem para a determinação do diâmetro da perfuração sob um diferente ponto de vista.

**Método:**

O diâmetro da perfuração septal, o diâmetro vertical total do septo e o diâmetro horizontal da perfuração foram medidos em de 34 pacientes; 19 foram submetidos ao reparo cirúrgico da perfuração septal e 15 receberam a aplicação do botão septal. Os pacientes foram solicitados a preencher o questionário de qualidade de vida *Glasgow Benefit Inventory*.

**Resultados:**

A perfuração septal cicatrizou com sucesso em 18 de 19 pacientes submetidos a tratamento cirúrgico. Os escores de qualidade de vida foram estatisticamente significativamente maiores no grupo de tratamento cirúrgico quando comparados aos do grupo que recebeu o botão septal (*p* < 0,05).

**Conclusão:**

A classificação de perfuração septal que fizemos seria benéfica para fornecer dimensões, métodos de tratamento e técnicas cirúrgicas realistas.

## Introduction

Septal perforation is a condition characterized by loss of cartilage and/or bony structures along with their mucoperichondrium and mucoperiosteum. The etiology includes history of nasal surgery or trauma, nose picking, bilateral septal cauterization, overuse of nasal sprays, cocaine abuse, vasculitis, and malignancies.^1^, ^2^ It is characterized by symptoms such as epistaxis, nasal clogging, nasal drainage, crusting, and wheezing.^3^

While septal perforation can be treated symptomatically with moisturizing creams and nasal irrigation, it can also be repaired with many surgical techniques described in the literature. It is possible to use prostheses such as septal buttons in patients who refuse surgical treatment or who are in an inappropriate general medical condition for surgery. The physician must consider the effects of symptoms in the patient's life while planning treatment. Additionally, there are authors who suggest that operating on asymptomatic cases is unnecessary.^4^

With this study, we aimed to bring a new viewpoint to the determination of the size of the septal perforation and also evaluate the quality of life in patients who have undergone surgery or received a septal button.

## Methods

### Choosing patients

Study protocol was approved from the Local Ethics Committee (15-KAEK-151). Between 2013 and 2016, 34 symptomatic patients complaining of epistaxis, nasal clogging, nasal drainage, crusting, and wheezing applied to our clinics. Nineteen patients underwent septal perforation repair using an external rhinoplasty technique. In addition, 15 patients with unsuitable conditions for surgery, refused the operation, and had a large perforation area, received septal button intervention under local anesthesia.

### Perforation size measurement method

**Figure 1:**
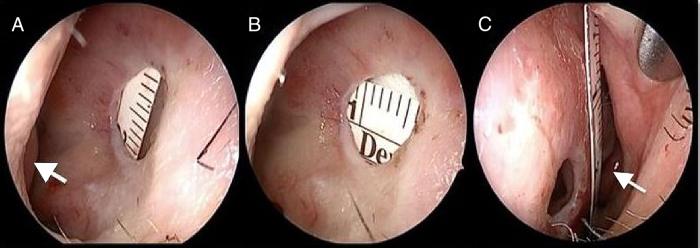
Measurement of vertical length (a), horizontal length (b) of septal perforation and measurement of vertical septal length (c). Short white arrow, inferior concha; long white arrow, left middle concha.

The diameters of perforation were measured at vertical (Fig. 1a) and horizontal (Fig. 1b) planes using a 30° endoscope with a tape measure. The total vertical length of the septum was measured again with a tape measure and a 30° endoscope (Fig. 1c). The septal perforations were categorized into four groups according to the ratio of the vertical length of septal perforation by the total vertical length of the septum.

Group 1 (small perforation) was defined as a length of perforation less than 1/4 of total vertical septal length.

Group 2 (medium perforation) was defined as length of perforation larger than 1/4 of total vertikal septal length and less than 1/2 of total vertical septal length.

Group 3 (large perforation) was defined as length of perforation larger than 1/2 of total vertical septal length and less than 3/4 of total vertical septal length.

Group 4 (very large perforation) was defined as length of perforation larger than 3/4 of total vertical septal length.

### Surgical procedure

**Figure 2:**
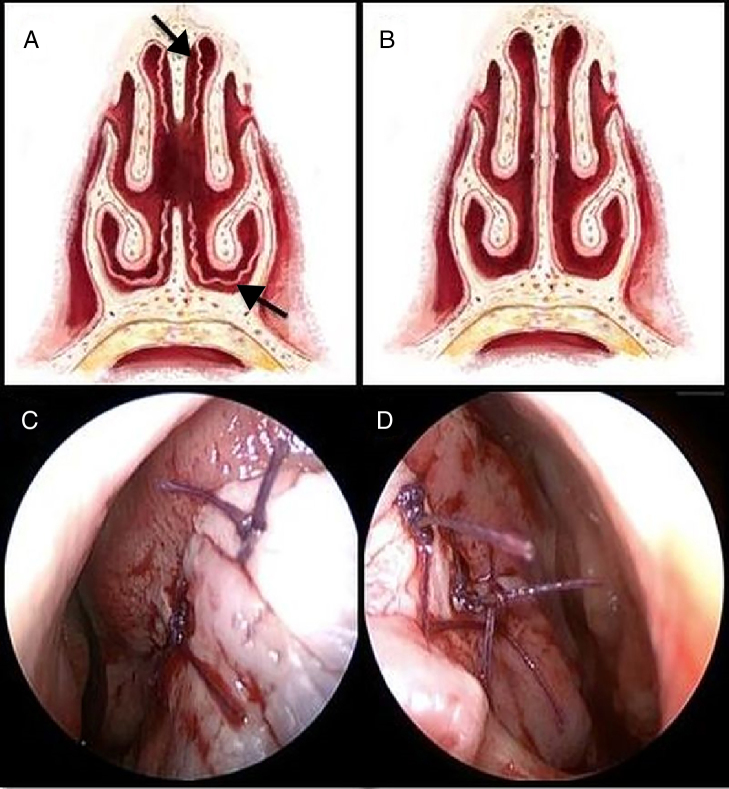
Schematic preparation of flaps (a), schematic suturing of flaps to each other (b), suturing of flaps on the right side (c), on the left side (d). Black Arrow, elevated mucosa.

**Figure 3:**
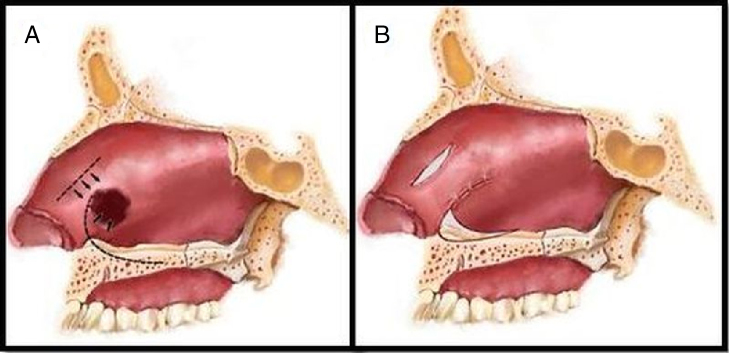
Preparation of flaps: lateral view (a) and suturing (b).

**Figure 4:**
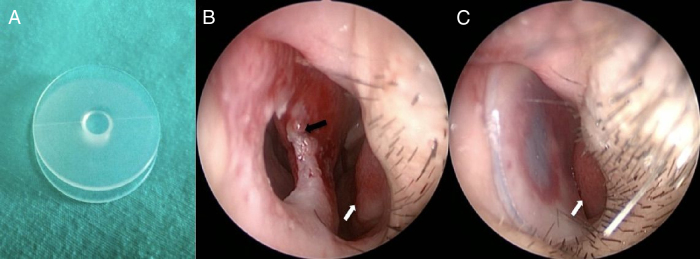
Septal button (a), septal perforation (b), patient with septal button (c). White arrows, inferior concha; black arrow, septal perforation.

All surgical procedures were performed under general anesthesia using an external rhinoplasty technique by two experienced surgeons. In order to anesthetize the skin of the nose, nasal septum, and borders of the perforation, 2% lidocaine with 0.0001% adrenaline were infiltrated. In order to prevent irregular lacerations during elevation of mucoperichondrium and mucoperiosteum, especially when there was insufficient cartilage and bone deposits around the perforation borders, the right and left nasal mucosa around the perforation borders were incised exactly at the middle line with a number 12 scalpel. The skin lining the nasal ridge was elevated by a “Goodman” incision on the columella.^5^ The caudal part of the septum was accessed between the medial crura of the alar cartilages. The mucoperichondrium and mucoperiosteum were elevated at both sides, up to the attachment site of inferior concha at the lateral border and roof of the nose at medial border (Fig. 2a). The structures causing deviation of the septum (if there were any) were corrected. After horizontal incisions parallel to the inferior concha beginning from the adhesion cite of the bilateral inferior concha, flaps were prepared using mucosa from the nose base. If the perforation could be repaired with this flap without tension, right and left mucoperichondrial and mucoperiosteal flaps were sutured to each other using 4/0 vicryl with a circular needle (Figs. 2 b–d). When minimal tension exists in patients, the mucosa under the upper lateral cartilage (ULC) was not elevated to preserve the nasal valve. In these cases a minimal length horizontal incision was made at the tension site and sutured (Figs. 3a–b). When the perforation was large and the tension was extreme, mucosa under the ULC was elevated and the borders of the perforation were sutured to each other (Fig. 2a). A 0° endoscope was used when the posterior side of the operational area could not be visualized during suturing. In order to increase stability and prevent recurring perforations, either septal cartilage or a few bone lamellae from perpendicular plate of ethmoid bone were placed at the suture line. The bone lamellae from the perpendicular plate of the ethmoid bone were only used when there was an insufficient amount of septal cartilage to be used. They were fixed with transseptal sutures. In order to increase the survivability of the flaps, silicone Doyle nasal splints (Osseous Rhinology Products, EON Meditech PVT LTD, India) were used instead of anterior tampons. These splints were kept in place for 10 days to protect the suture site against external factors as much as possible. Septal button (Silicone Septal-Button, Spiggle&Theis, Medizintechnik, Germany) insertion under local anesthesia was preferred in patients who were not suitable for surgery or who refused to be operated on again, and in whom (Group 4) surgical treatment might not completely repair the perforation site (Fig. 4).

### Quality of life questionnaire

Following the control follow-up examination in the 1st month, all patients were asked to come for an additional examination in the 2nd month in order to standardize the follow-up program. Patients who accepted the invitation completed the questionnaire by themselves and patients who did not accept fulfilled the questionnaire via a phone call.

Glasgow benefit inventory (GBI) is sensitive tool for identifying changes in health status after an operation. The GBI consists of 18 questions that assess different aspects of health-related quality of life.^6^ Answers are chosen from a 5 level Likert scale ranging from 1 to 5. The points contain the GBI score, which ranges from −100 (adverse effect), 0 (no effect), to +100 (maximal positive effect). After completion of the questionnaire, total score and subscores, including overall score, social support score, and physical health score, can be calculated.^7^, ^8^

Data were analyzed using SPSS version 15, and Mann–Whitney *U* and Wilcoxon tests were used for comparing GBI scores of the two groups. The findings were considered significant when *p* was <0.05.

## Results

Demographic data of the 19 patients who underwent perforation repair and the 15 patients who had septal button insertion are listed in Table 1.

**Table 1:** Demographic data of groups.

	Age groups				Perforation groups			
	<20	21–40	41–60	>60	Group 1	Group 2	Group 3	Group 4
*Operated group*								
Male	–	6	1	–	3	3	2	–
Female	4	4	4	–	2	5	4	–

*Button insertion group*								
Male	–	4	3	–	–	1	4	2
Female	1	1	4	2	–	–	4	4

The follow-up periods were between 6 and 20 months, with a mean of 13.3 ± 2.92 months. Seven of the patients underwent septoplasty, two underwent nasal valve surgery, and four underwent nasal tipplasty in the same session.

During mucoperichondrial elevation, even though no mucosal damage had been done, the diameter of the perforation increased in patients undergoing septal perforation repair. Bilateral inferiorly based flaps were used in all patients who underwent surgery. A 1.5 cm-long horizontal mucosal incision at the level of the nasal valve was made in only five of these patients because of flap tension. The septal perforation was repaired with flaps based at both superior and inferior parts in six patients by elevating the mucosa under the upper lateral cartilage. The perforation could not be repaired completely in one patient, and a septal button was inserted two months later. This patient was in Group 3 and had undergone submucosal resection previously. The mucoperichondrial and mucoperiosteal flaps were quite thin. The symptoms of patients before and after the operation and septal button insertion are listed in Table 2.

**Table 2:** Patients’ scale of symptoms before and after the operation and septal button insertion.

Symptoms	Operated group			Septal button insertion group		
	Before	After	% Improvement	Before	After	% Improvement
Bleeding	10 (57%)	1 (5%)	90	9 (60%)	2 (13%)	77
Whistling	5 (26%)	–	100	–	–	–
Crusting	10 (57%)	–	100	11 (73%)	10 (66%)	9
Stenosis	6 (31%)	5 (26%)	83	10 (66%)	4 (26%)	60
Foul smell	6 (31%)	–	100	4 (26%)	8 (53%)	0
Sneezing	8 (42%)	8 (42%)	0	7 (46%)	7 (46%)	0

Only one of our patients experienced epistaxis on postoperative 3rd day. It originated from the incision site at the superior part and was stopped with electrocautery. Nasal valve stenosis was observed bilaterally in two and unilaterally in one of six patients who had superiorly based flaps. These patients underwent nasal valve surgery six months later. The closure rates in the first month did not change during the follow-up period. Endoscopic examination at least two months after the operation revealed complete epithelization of the flaps. The perforation was repaired completely in 18 of 19 patients.

The total score of the GBI was 28.7 in the operated group (OG) and 17.9 in the septal button group (SBG), showing benefit from the operation. The general subscale score was 42.6 (OG) and 22.8 (SBG), social support score was 21.4 (OG) and 14.8 (SBG), and physical health score was 11.5 (OG) and 8.1 (SBG). According to these scores, patients’ health-related quality of life scores increased after the operation in both groups. When health-related quality of life scores of OG and SBG were compared, a statistically significant difference was found. OG had statistically significantly higher health-related quality of life scores (Table 3).

**Table 3:** Quality of life scores.

	Total score (min–max)	General subscale score (min–max)	Social support score (min–max)	Physical health score (min–max)
Operated group	28.7 (17–40)	42.6 (26–50)	21.4 (15–32.5)	11.5 (5.5–20)
Buton insertion group	17.9 (5–28)	22.8 (5–35)	14.8 (5–35)	8.1 (5–12)
*p*	<0.05	<0.05	<0.05	<0.05

## Discussion

Functionally, nasal septal perforations may cause breathing difficulties and distorted nasal air transport and esthetically, retraction of the columella and saddle nose deformity.^9^ Along with these, complaints such as wheezing with small perforations and dryness and bleeding with large perforations are also quite important.^10^ Since the most frequent etiologies are submucosal resection and septoplasty, when lacerations are noticed at opposing mucoperichondrial and/or mucoperiosteal flaps, they must be immediately sutured to prevent further septal perforation, which is difficult to treat. The treatment may not yield satisfactory results in perforations caused by cocaine abuse, granulomatous diseases, nose picking habit, and cauterization.^11^ The septal perforation failed to completely heal in only one of our patients, and the higher quality of life scores in surgical group indicates that we had satisfactory results. Septal button insertion in patients who didn’t benefit from surgical treatment increases incrustation, but, on the other hand, it is an alternative treatment that reduces epistaxis, wheezing, and nasal awareness symptoms.^12^ Our patients also experienced partial improvements of these symptoms.

Most patients with septal perforation are asymptomatic.^13^ The perforation is located at the anterior cartilage part of the septum in those who are symptomatic.^2^ Eighteen of our patients had perforations located at the anterior part of the septum.

There is no available classification regarding the size of the septal perforation.^11^, ^14^, ^15^ In the surgical method we used, the ultimate determinant of perforation repair is the vertical size of the perforation and the presence of adequate septal tissue to cover the defect. Therefore, our surgical technique is appropriate for Groups1–3 and may not be appropriate for Group 4 patients. So we did not apply this technique to any of the patients in Group 4. Past cadaveric studies found the vertical size of the septum to vary significally.^16^, ^17^ Thus, we think that the vertical size of the septum and the vertical size of the perforation must be measured in every patient when determining the size of the perforation. The ratio of these two measurements could help us determine a more realistic perforation size. While it can be very easy to repair a 2 cm long perforation in a patient with a 3.5 cm long septal vertical diameter, it can be rather difficult to repair a 2 cm long perforation in a patient with a 2.5 cm septal vertical diameter. In order to prevent this complicated situation, we consider that evaluating the perforation size individually according to each patient would be more appropriate. Thus, we propose a classification for determining the size of the perforation. We measured the widest vertical diameter of the perforation and vertical length of the septum in the plane where the perforation is widest. By comparing these two measurements, we created a classification consisting of four groups. We think that this new classification could help surgeons to determine precise sizes of the septal perforations before surgery, choose treatment methods, determine the appropriate surgical technique, and predict surgical success rates.

Septal perforation can be treated surgically or in a more conservative non-surgical manner. Nasal irrigation, ointments, and silicone septal buttons might be suggested with conservative treatment in order to prevent conditions like epistaxis and crusting. Septal buttons may also cause foreign body reaction in the nose and might increase inflammation and incrustation.^18^, ^19^ In order to prevent these situations, patients must be treated with the most appropriate method simulating normal physiology. Studies suggest that septal buttons might be used as an alternative treatment in patients that could not be operated on. They improve symptoms in most patients but cannot provide a precise cure. Thus, some patients ask for the prostheses to be removed after a short while. The symptoms that affect them least are crusting, congestion, pain, and runny nose.^20^ Consistent with the literature, the most common complaints among our patients were incrustation, congestion, and runny nose.

Many techniques have been reported in the literature for surgical treatment; however, surgeons still struggle for a successful treatment. There is no consensus on the method to be used.^21^, ^22^, ^23^ The most important factors affecting the success of surgery are size and location of the septal perforation, amount of tissue left at the septum, tension of the flaps, and experience of the surgeon.^24^, ^25^, ^26^ It is possible to repair septal perforations by external rhinoplasty, closed endonasal, and even endoscopic methods. The disadvantages of the closed endonasal technique are the limited field of view and difficult suturing. External technique provides both better field of view and easy suturing.^24^ We operated on all of our cases using the external rhinoplasty method. We used an endoscope for suturing in cases with perforation extending to the posterior part of the septum. The success rate of closed endonasal and open perforation repairing methods does not differ a lot.^27^, ^28^ The location and size of the perforation, alongside habits and preferences of the surgeon, determine the treatment method to be used. Placing autografts (bone or cartilage) between the flaps for supportive purposes has been reported to heal 2–3 cm long perforations with 90% success rate.^28^ In addition to these tissues, use of fascia of temporal muscle, conchal cartilage, and alloderm have been reported in the literature.^10^, ^29^ In fact, it has been reported in the literature that it is possible to completely cover the perforation site with polyethylene implants and the mucosa will advance over this implant to heal the perforation.^30^ We used septal cartilage or perpendicular plate of ethmoid bone (when septal cartilage was not available) in our cases and achieved a 95% success rate, which is consistent with the literature. We think that the problem in the patient who cannot close the perforation is originating from impaired nutrition.

Although the survey scores for quality of life were not much different in patients, who underwent septoplasty and nasal valve surgery compared to the patients, who underwent only a repairment of the nasal septal perforation, the scores of the patients who underwent nasal tip plasty were relatively higher. We believe that this finding suggests that only the breathing was improved in patients who underwent nasal valve surgery and septoplasty, but in patients, who underwent nasal tip plasty, there was also an additional nasal cosmetic improvement.

Higher GBI scores of patients treated with mucoperichondrial and/or mucoperiosteal flaps when compared to patients treated with septal buttons are supportive of the Gillies’ theory, which is the principle that the area where the tissue loss exists must be treated with similar tissues.^31^ Our main surgical goal when repairing septal perforations is not only covering the perforation but also maintaining nasal physiology and normal function, which is most appropriately achieved by using local flaps.

We used the GBI scale to objectively evaluate the patients’ quality of life, as in the literature. The questions of these scales were prepared to determine the health-related quality of life of the patient retrospectively, especially following plastic, reconstructive and/or otolaryngology surgeries. These scales were selected for the most sensitive identified cases affecting health-related quality of life.^8^ The data we obtained from our patients support that the patients benefited from the procedures we used. We consider that higher GBI scores of the surgical group are because of the fact that surgical closure of the perforation brings nasal physiology back to its normal function.

## Conclusion

Since the most common etiologic cause of nasal septal perforations is septoplasty, perforations that occur during the operation must be closed with primary suturing. We developed a new way to classify septal perforations. It helps to determine which technique to choose to close the perforation, helped us to have a 95% of success and this study showed surgery is better than septal buttons according to the both post-operation follow-up visits and quality of life questionnaires. Patients cannot experience recovery in their quality of life unless the normal nasal physiology is established. Because of the high success rate of the surgical treatment, it should strongly be considered in patients requiring perforation repair.

## Conflicts of interest

The authors declare no conflicts of interest.

## References

[1] Meyer R. (1994). Nasal septal perforations must and can be closed. Aesthetic Plast Surg Fall.

[2] Cogswell L.K., Goodacre T.E. (2000). The management of nasoseptal perforation. Br J Plast Surg Mar.

[3] Wong S., Raghavan U. (2010). Outcome of surgical closure of nasal septal perforation. J Laryngol Otol.

[4] Tardy M.E. (1977). Practical suggestions on facial plastic surgery-how I do it Sublabial mucosal flap: repair of septal perforations. Laryngoscope.

[5] Goodman W.S., Charbonneau P.A. (1974). External approach to rhinoplasty. Laryngoscope.

[6] Braun T., Hainzinger T., Stelter K., Krause E., Berghaus A., Hempel J.M. (2010). Health-related quality of life, patient benefit, and clinical outcome after otoplasty using suture techniques in 62 children and adults. Plast Reconstr Surg.

[7] Toplu Y., Sapmaz E., Firat C., Toplu S.A. (2014). Clinical results and health-related quality of life in otoplasty patients using cartilage resection and suturing methods. Eur Arch Otorhinolaryngol.

[8] Robinson K., Gatehouse S., Browning G.G. (1996). Measuring patient benefit from otorhinolaryngological surgery and therapy. Ann Otol Rhinol Laryngol.

[9] Ribeiro J.S., da Silva G.S. (2007). Technical advances in the correction of septal perforation associated with closed rhinoplasty. Arch Facial Plast Surg.

[10] Andre R.F., Lohuis P.J., Vuyk H.D. (2006). Nasal septum perforation repair using differently designed, bilateral intranasal flaps, with nonopposing suture lines. J Plast Reconstr Aesthet Surg.

[11] Younger R., Blokmanis A. (1985). Nasal septal perforations. J Otolaryngol.

[12] Kridel R.W. (1999). Septal perforation repair. Otolaryngol Clin North Am.

[13] Brain D.J. (1980). Septo-rhinoplasty: the closure of septal perforations. J Laryngol Otol.

[14] Goh A.Y., Hussain S.S. (2007). Different surgical treatments for nasal septal perforation and their outcomes. J Laryngol Otol.

[15] Metzinger S.E. (2005). Diagnosing and treating nasal septal perforations. Aesthet Surg J.

[16] Mowlavi A., Masouem S., Kalkanis J., Guyuron B. (2006). Septal cartilage defined: implications for nasal dynamics and rhinoplasty. Plast Reconstr Surg.

[17] Miles B.A., Petrisor D., Kao H., Finn R.A., Throckmorton G.S. (2007). Anatomical variation of the nasal septum: analysis of 57 cadaver specimens. Otolaryngol Head Neck Surg.

[18] Osma U., Cureoglu S., Akbulut N., Meric F., Topcu I. (1999). The results of septal button insertion in the management of nasal septal perforation. J Laryngol Otol.

[19] Beekhuis G.J., Eisenstein B. (1977). Repair of nasal septal perforation with a silicone button. Laryngoscope.

[20] Dosen L.K., Haye R. (2008). Silicone button in nasal septal perforation Long term observations. Rhinology.

[21] Cassano M. (2014). Endoscopic repair of nasal septal perforation with “slide and patch” technique. Otolaryngol Head Neck Surg.

[22] Schultz-Coulon H.J. (1997). Nasal septum repair-plasty with pedicled flap technique in 126 patients—an analysis. Laryngorhinootologie.

[23] Castelnuovo P., Ferreli F., Khodaei I., Palma P. (2011). Anterior ethmoidal artery septal flap for the management of septal perforation. Arch Facial Plast Surg.

[24] Parry J.R., Minton T.J., Suryadevara A.C., Halliday D. (2008). The use of fibrin glue for fixation of acellular human dermal allograft in septal perforation repair. Am J Otolaryngol.

[25] Tastan E., Aydogan F., Aydin E., Can I.H., Demirci M., Uzunkulaoglu H. (2012). Inferior turbinate composite graft for repair of nasal septal perforation. Am J Rhinol Allergy.

[26] Teymoortash A., Hoch S., Eivazi B., Werner J.A. (2011). Experiences with a new surgical technique for closure of large perforations of the nasal septum in 55 patients. Am J Rhinol Allergy.

[27] Fairbanks D.N. (1980). Closure of nasal septal perforations. Arch Otolaryngol.

[28] Kridel R.W., Appling W.D., Wright W.K. (1986). Septal perforation closure utilizing the external septorhinoplasty approach. Arch Otolaryngol Head Neck Surg.

[29] Kridel R.W., Foda H., Lunde K.C. (1998). Septal perforation repair with acellular human dermal allograft. Arch Otolaryngol Head Neck Surg.

[30] Cho J.J., Taylor R.C., Deutschmann M.W., Chandarana S.P., Marck P.A. (2013). Polyethylene implants in nasal septal restoration. JAMA Facial Plast Surg.

[31] Gillies H. (1957).

